# The Utility of Cloud Computing in Analyzing GPU-Accelerated Deformable Image Registration of CT and CBCT Images in Head and Neck Cancer Radiation Therapy

**DOI:** 10.1109/JTEHM.2016.2597838

**Published:** 2016-08-17

**Authors:** George Zaki, William Plishker, Wen Li, Junghoon Lee, Harry Quon, John Wong, Raj Shekhar

**Affiliations:** 1 IGI Technologies, Inc.College ParkMD20742USA; 2 Radiology and Biomedical Imaging DepartmentUniversity of California at San Francisco 8785 San FranciscoCA94115USA; 3 Department of Radiation Oncology and Molecular Radiation SciencesThe Johns Hopkins School of MedicineThe Johns Hopkins University 1466 BaltimoreMD21231USA; 4 Sheikh Zayed Institute for Pediatric Surgical InnovationChildren’s National Medical Center 8404 WashingtonDC20010USA

**Keywords:** Cloud computing, computed tomography, image registration, oncology, parallel programming

## Abstract

The images generated during radiation oncology treatments provide a valuable resource to conduct analysis for personalized therapy, outcomes prediction, and treatment margin optimization. Deformable image registration (DIR) is an essential tool in analyzing these images. We are enhancing and examining DIR with the contributions of this paper: 1) implementing and investigating a cloud and graphic processing unit (GPU) accelerated DIR solution and 2) assessing the accuracy and flexibility of that solution on planning computed tomography (CT) with cone-beam CT (CBCT). Registering planning CTs and CBCTs aids in monitoring tumors, tracking body changes, and assuring that the treatment is executed as planned. This provides significant information not only on the level of a single patient, but also for an oncology department. However, traditional methods for DIR are usually time-consuming, and manual intervention is sometimes required even for a single registration. In this paper, we present a cloud-based solution in order to increase the data analysis throughput, so that treatment tracking results may be delivered at the time of care. We assess our solution in terms of accuracy and flexibility compared with a commercial tool registering CT with CBCT. The latency of a previously reported mutual information-based DIR algorithm was improved with GPUs for a single registration. This registration consists of rigid registration followed by volume subdivision-based nonrigid registration. In this paper, the throughput of the system was accelerated on the cloud for hundreds of data analysis pairs. Nine clinical cases of head and neck cancer patients were utilized to quantitatively evaluate the accuracy and throughput. Target registration error (TRE) and structural similarity index were utilized as evaluation metrics for registration accuracy. The total computation time consisting of preprocessing the data, running the registration, and analyzing the results was used to evaluate the system throughput. Evaluation showed that the average TRE for GPU-accelerated DIR for each of the nine patients was from 1.99 to 3.39 mm, which is lower than the voxel dimension. The total processing time for 282 pairs on an Amazon Web Services cloud consisting of 20 GPU enabled nodes took less than an hour. Beyond the original registration, the cloud resources also included automatic registration quality checks with minimal impact to timing. Clinical data were utilized in quantitative evaluations, and the results showed that the presented method holds great potential for many high-impact clinical applications in radiation oncology, including adaptive radio therapy, patient outcomes prediction, and treatment margin optimization.

## Graphical abstract

The images generated during radiation oncology treatments provide a valuable resource to conduct analysis for personalized therapy, outcomes prediction, and treatment margin optimization. Deformable image registration (DIR) is an essential tool in analyzing these images. This work presents: (1) implementing and investigating a cloud and graphic processing unit (GPU) accelerated DIR solution and (2) assessing the accuracy and flexibility of that solution on planning computed tomography (CT) with cone-beam CT (CBCT). Results showed that the presented method holds great potential for many high-impact clinical applications in radiation oncology including adaptive radio therapy, patient outcomes prediction, and treatment margin optimization.

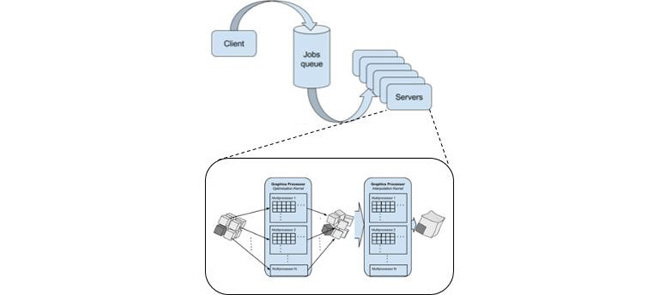

## I. Introduction

Patient positioning and immobilization are crucial to ensuring accurate dose delivery to tumors, with minimal damage to surrounding normal tissues. However, geometric uncertainty still exists [1], and, to account for it, margins are added to the actual tumor boundaries at the time of planning. The challenge of delineating the margin is to ensure enough treatment coverage of the tumor while reducing normal tissue damage. This challenge is acute in head and neck patients because the tumor is more likely to be surrounded by critical organs, such as the spinal cord, brain, eyes, ears, and parotid glands. Fortunately, cone beam CTs (CBCTs) that are used for positioning can track some of these changes. As these CBCTs are taken at every fraction, dozens of images are generated for each patient creating a need to processes them with high throughput and accurate algorithms. Furthermore, automatic assessment, and reduction of manual guidance are two key requirements for such systems. A fully automatic, deformable image registration (DIR) of CBCTs to planning CTs is a foundational tool for tracking body changes to ultimately generate this information about patients or hospitals. By accelerating DIR with graphics processors (GPUs) and cloud computing, we enable the sort of high throughput necessary to create this information at the time of treatment.

The most commonly used imaging modality for planning radiation therapy is CT. After planning, the treatment is completed in several daily fractions that together can last up to two months. CBCT plays a major role in repositioning the patient for daily treatment fractions. With CBCT, a 3D scan of the patient on the treatment couch is taken immediately before daily treatment. The CBCT is reconstructed and the patient is repositioned based on the registration of the CBCT with the planning CT. Rigid registration is often used in this scenario because it is fast and sufficient for generating a couch shift (i.e., 3D translational and/or rotational shift). However, this mode of registration does not account for nonrigid anatomical changes from the time of planning CT.

Nonrigid changes in radiation oncology patients may originate from various sources such as weight change, tumor shrinkage, etc. Such changes can cause deviation from the original treatment plan due to over or under treatment of structures. Plans do provision for such changes, but this is often at the cost of overly conservative treatment margins. If these changes could be detected through the use of DIR, it would be possible to account for these changes, enabling margin optimization studies. DIR generates a unique displacement for each pixel/voxel of the floating image such that they align with the corresponding pixel/voxel of the reference image. However, a limitation of DIR is that it is computationally complex and therefore usually slow.

DIR has a number of applications in radiation therapy [2], [3]. In almost all of these applications, it is utilized to track nonrigid anatomical changes from the time of treatment planning to the time of daily treatment.

The focus of this article is to show the utility of GPUs and cloud computing in speeding up the image analysis and the generation of specialized information (i.e., on the personal or department level) in the context of radiation therapy. We evaluate the underlying DIR algorithm on representative clinical data because the algorithm must be accurate and fast and capable of massive data processing for carrying out the DIR-enabled studies effectively and efficiently.

## II. Related Work

A comprehensive review of DIR methods in radiation therapy can be found in the literature [3]. There exist two principal DIR approaches: feature-based and intensity-based. Compared with feature-based DIR, intensity-based DIR, our preferred approach, does not rely on feature detection and feature matching. It searches for an optimal transformation between two images based on image intensities instead. Although capable of being fully automated and arguably more accurate, intensity-based DIR is usually slower than feature-based DIR. In the family of intensity-based DIR methods, traditional Demons and its variants utilize optical flow-based strategy to deform one image to align with the other [4]. These methods have a range of applications in radiation therapy because of Demons algorithm’s speed and simplicity [5]–[7]. However, these methods modify CBCT intensity values to reduce intensity deviations between CT and CBCT because Demons algorithm assumes that the two images to be registered are of the same modality. Although based on the same physical principles with conventional CT, CBCT does not yield a consistent intensity value for a particular tissue type because of the underlying Feldkamp reconstruction algorithm generates a high-quality image only at the central plane and intensity degradation increases linearly with the distance from it [8], [9]. In addition, noise and artifacts due to small field of view and hardware limitations in CBCT also cause intensity deviation between CBCT and conventional CT [10].

Mutual information (MI) is the most effective currently known image similarity measure for multimodality DIR [11], [12]. A popular MI-based DIR algorithm based on free-form deformation was proposed by Rueckert et al. [13]. In this algorithm, B-splines are used to describe the smooth and continuous free-form deformation. This method is particularly suitable for recovering local deformations but its accuracy relies highly on the density of the control points, and the computation time increases exponentially with the number of control points.

CT-to-CBCT registration using MI- and B-spline-based DIR has been reported, an example of which is the method reported by Paquin et al. [14]. These investigators further used a multiscale approach to improve the method’s efficiency for CT-CBCT registration. The radiation oncology-oriented commercial software, Velocity Advanced Imaging (Velocity Medical Solutions, Atlanta, GA), offers B-spline-based DIR with MI as the image similarity measure as part of its fusion module. The Velocity DIR has been evaluated with both phantom and clinical images [15], [16]. In clinical validations, Lawson et al. focused on its applicability to CT-CBCT registration [16]. Both studies showed that the Velocity DIR algorithm was accurate and robust to noise in CBCT images. While accurate, commercial software such as Velocity Medical’s grants limited accessibility and flexibility to users for making application-specific customizations and seamless integration into other software workflows.

Distributed computing used for image registration has also been explored previously. Image registration was used as the driver of an investigation into virtual computational cloud that integrates local computational environments and public cloud services on-the-fly, and support image registration requests from different distributed research groups [17]. Grid computing has also been applied to image registration by viewing the problem as a mesh and decomposing it [18]. We have also previously investigated the use of cluster computing in the context of image registration. By controlling network topology and fine grain scheduling, subtasks of a single registration may be farmed out to local nodes [19].

In this article, we improve upon the previous work by using a graphics processing unit (GPU)-accelerated hierarchical volume subdivision (VS)-based DIR method in a GPU-enabled cloud environment. In contrast to the previous work on distributed computing, a cloud computing image registration solution exists on a spectrum between a grid and a cluster. Resources of a grid can be spread across geography and even different owners, while clouds are typically centrally managed and located, providing an opportunity to overcome transmission latency limitations reported in [18]. At the other end of the distributed spectrum, clusters typically operate on a local area network capable of providing fine grain control of scheduling. Clouds by contrast use nodes which are loosely coupled. By decoupling the computation for a cloud implementation, there is an opportunity to provide more scalability and less complexity than our previous cluster based solution [19]. The core algorithm was developed by our team and has been reported and extensively validated previously [20]–[23]. This implementation is referred to as “VS-GPU” henceforth in this article. We further present a quantitative evaluation of applying the VS-GPU algorithm to the registration of clinical CT-CBCT images of head and neck cancer patients and demonstrate that both its speed and accuracy are acceptable for use in radiation oncology studies.

## III. Methods

### A. Data Acquisition

Archived images of 9 patients who underwent radiation treatment for head and neck cancer were chosen for the current study. The use of human subject data was approved by the Institutional Review Board (IRB) of Johns Hopkins Hospital and IGI Technologies. Each patient had one planning CT and a set of CBCTs, one for each treatment fraction typically coinciding with a daily visit. Characteristics of 9 patients’ imaging data are presented in Table 1. The entire dataset was divided into a training set (Cases 1–4) and a test set (Cases 5–9). The training set was used to select optimal parameters for VS-GPU and the test set was used to evaluate it.

**TABLE 1:** Characteristics of Imaging Dataset for 9 Head and Neck Cancer Patients

Case Number	# of Fractions	# of Slices	Voxel Size (mm^3^)	Couch Shift (mm)
Case 1	35	137	$1.3\times 1.3\times3.0$	3.4 ± 1.2
Case 2	32	150	$1.4\times 1.4\times3.0$	2.3 ± 1.3
Case 3	32	146	$1.2\times 1.2\times3.0$	4.4 ± 2.2
Case 4	33	161	$1.2\times 1.2\times3.0$	4.3 ± 0.9
Case 5	48	146	$1.3\times 1.3\times3.0$	2.2 ± 1.0
Case 6	31	149	$1.3\times 1.3\times3.0$	3.1 ± 1.2
Case 7	28	134	$1.2\times 1.2\times3.0$	3.4 ± 0.9
Case 8	30	146	$1.2\times 1.2\times3.0$	4.4 ± 1.4
Case 9	24	129	$1.2\times 1.2\times3.0$	2.2 ± 1.1

Planning CTs were acquired on a Philips Brilliance Big Bore CT scanner (120 kVp; 413 mA; 600 mm Field of View; 3.0 mm thickness; Philips Medical Systems, Cleveland, OH) and had an image size of $512 \times 512 \times 129$–161 voxels (see Table 1). CBCT images were acquired 1–10 weeks after the acquisition of the planning CT with an on-board Elekta Synergy XVI cone beam CT (Elekta Inc., Maryland Heights, MO) during each fraction that numbered one or two a day. CBCT images were resampled and zero-padded to have the same image dimensions as those of the planning CT scan and then exported as DICOM images for each patient. The number of CBCTs varied from 16 to 48 for a given patient.

Along with the CBCT images, we also obtained the dose calculation point (CALC) and the couch shift applied to reposition the patient during each treatment fraction from MOSAIQ system (Elekta Inc., Maryland Heights, MO). CALC is a reference point where the radiation dose is documented and monitored. It is usually placed at the center of the planned tumor volume (PTV) or coincides with an anatomical landmark within PTV. The couch shift was recorded as a 3D translational shift vector, comprising the shift of the treatment couch in left-to-right, anterior-to-posterior, and superior-to-inferior directions. The vector length was considered as the amount of couch shift, which ranged between 0.0 mm-11.6 mm for our dataset. The average couch shift (averaged over all fractions) for each case is shown in Table 1.

### B. Registration Framework

As reported, we have found the VS-based DIR algorithm with MI as a similarity measure effective in multimodality image registration [20]–[23]. We extended our previous work to add GPU acceleration to the algorithm for speed and therefore suitable for clinical use, while maintaining the accuracy of the original algorithm.

**FIGURE 1.**
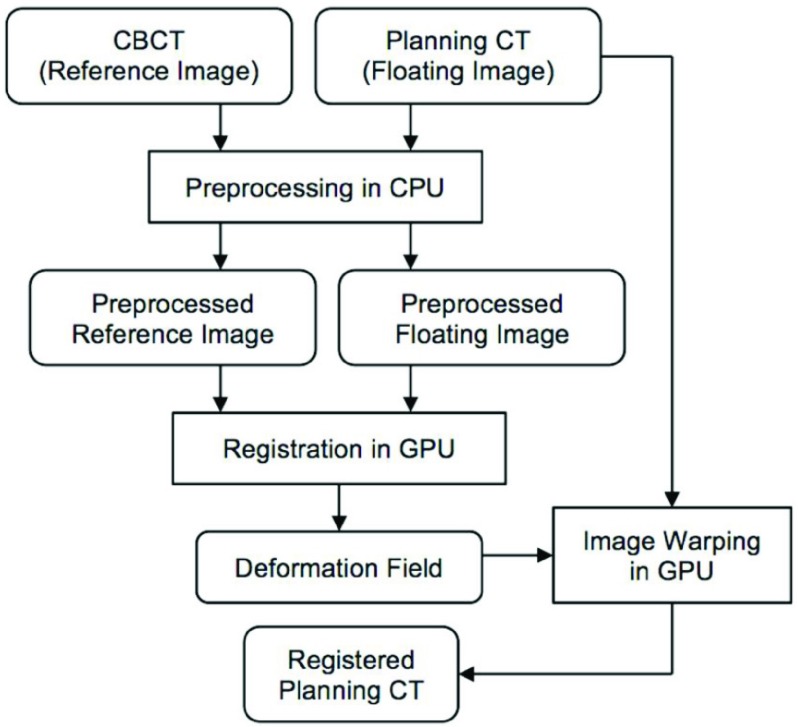
The flowchart of GPU-accelerated deformable registration of planning CT and CBCT.

The framework of the VS-GPU applied in CT-CBCT registration is shown in Figure 1, and it is used to register the planning CT (the floating image) with each daily CBCT (the reference image) of the same patient. First, each input image is rescaled by a preset window/level determined by our parameter optimization (see Section D). A 3D discrete Gaussian smoothing with voxel-size variance in each direction is then applied to both reference and floating images. Next, the preprocessed images are nonrigidly registered on a Dell Alienware Aurora R4 desktop PC with dual NVidia GeForce GTX 680 graphic cards.

The volume subdivision-based DIR algorithm is a locally rigid but globally nonrigid registration algorithm. MI is the similarity measure in the VS-based registration at all levels and for all registration tasks and is calculated using the histogram method [11], [12]. The downhill simplex method is used to search for the optimal MI value between the images for all registration tasks. The reader is referred to our previous work for details of the algorithm [20].

**FIGURE 2.**
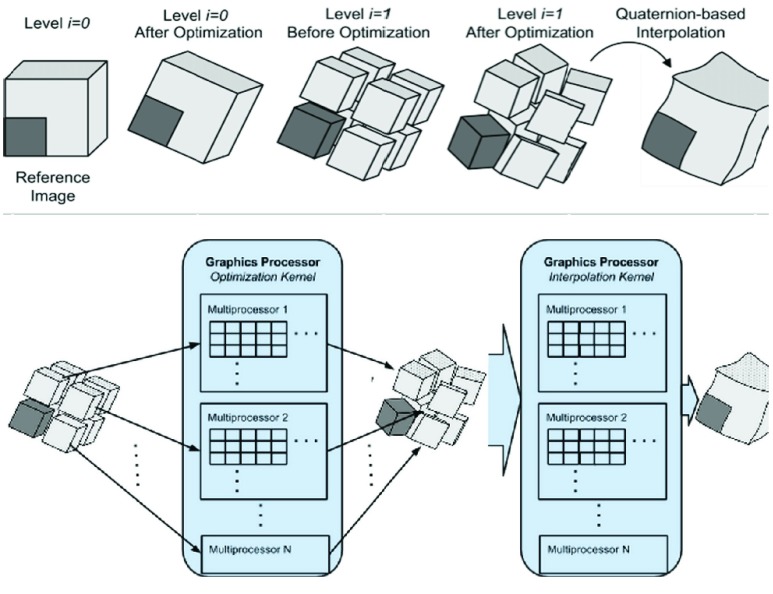
The concept of volume subdivision to achieve DIR and how it maps to a GPU.

Volume subdivision is performed on grids of the reference image based on hierarchical octree. At first, the reference image is divided into eight equal subvolumes, and the original (global) transformation of each of the eight subvolumes with the floating image is refined individually by rigid registration. In the next pass, each of the subvolumes is split into eight subvolumes of their own, and the orientation of each of the child subvolumes is refined as well. Figure 2 shows the subdivision concept. The process continues until the subvolumes reach a threshold size (for example, $16 \times 16 \times 8$). Each subvolume registration is constrained by the maximum allowable displacement for a given subdivision level to avoid image-folding artifacts and preserve image topology. After all the subvolumes at the finest level of the subdivision are registered, the final deformation field is generated by quaternion-based cubic interpolation of individual rigid transformations.

The structure of our algorithm lends itself to efficient implementation on a GPU. While a GPU can be an inexpensive, low-power consumption platform of high computational throughput, it is capable of these feats when applications map well to its overall architecture. Applications that map well are those that can be decomposed into independent blocks of computations that may be further decomposed into coordinated light-weighted threads. Such an application decomposition is well tailored to many cores of a GPU and quite different from parallelization strategies employed for traditional high performance computing, which encourage constructing a homogenous set of heavy-weight threads.

In VS-GPU, we utilize NVIDIA’s Compute Unified Device Architecture (CUDA) to map each subvolume to a block of threads (CUDA block) and each voxel in a subvolume to a thread (CUDA thread) as shown in Figure 2. The calculation of similarity measure (i.e., MI) within a subvolume is independent from other subvolumes, mapping well to restrictions on CUDA blocks. Threads themselves are tasked with looking up reference and candidate floating image voxel intensities and updating mutual histogram. Unlike CUDA blocks, CUDA threads within a block are able to coordinate closely to efficiently load neighborhood image data, and coordinate updates to the local mutual histograms. This matching of the application to architecture results in orders of magnitude acceleration of the application, with no changes to the algorithm itself.

### C. Registration Evaluation Metrics

The performance of our VS-GPU registration was evaluated in comparison with the peer software applied to the same clinical data. We used Target Registration Error (TRE) as the metric for evaluation and structural similarity (SSIM) index.

Clinically documented points combined with couch shifts were used to calculate TRE in this paper. CALC is a point whose coordinates are documented in the planning CT for prescribing, recording, and monitoring radiation dose. It is usually inside the PTV or coincides with an anatomic landmark. Any repositioning strategy taken in daily radiation treatment focuses on precise alignment of this particular point. Thus, the CALC in planning CT when applied with the inverse couch shift should produce the same point in the prepositioned CBCT.

SSIM index was utilized as a second metric to measure the similarity between CT and CBCT based on the whole 3D image volume. Derived from the universal quality index (UQI), SSIM is originally a statistical image quality assessment index composed of three components–luminance, contrast, and structure–that simulate human vision system to detect changes in object structures that can be used to measure structural similarity between two images [24]. A major difference with respect to other similarity measures such as MI and cross correlation (CC), which are also eligible for measuring similarity between multimodality images, is that SSIM index was designed to detect perceived change in structural information.

Computation time was used to evaluate the speed of registration. It was calculated as the total elapsed time of DIR, excluding the preprocessing (approximately 10 s in CPU) and the final warping to generate the registered image (up to 2 s in CPU). Both VS-GPU and Elastix (a peer software described in Section E) were recorded on the same computer while Velocity registration software was run on a desktop computer with Intel Xeon Processor @2.40GHz and 12GB RAM.

### D. Cloud Acceleration

In addition to GPU acceleration, all the processing tasks to preprocess, run, and evaluate our algorithm were conducted on an AWS cloud using GPU enabled compute nodes. The cloud system consists of:

1- Amazon Elastic Compute Cloud (EC2) nodes. These nodes are preconfigured with our registration software, preprocessing tools, and evaluation scripts. The number of nodes to be launched can be set at runtime. In our evaluation we set this number to 20 nodes.
2- An Amazon Simple Storage System bucket: The bucket holds all the anonymized patient images and planning contours. There is a one time setup for this bucket to populate it with images. Once the images are on the cloud, there is a minimal cost in terms of communication time to move these data to the EC2 nodes for processing.
3- An Amazon Simple Queue Systems (SQS): The SQS can hold an unlimited number of messages where every message has a payload of 256KB. The queue contains a number of messages equal to the number of registration pairs. We configured the payload of the these message to include the input data that will be copied from the S3 bucket, the executables to run, and the output files that will be copied back to the S3.

**FIGURE 3.**
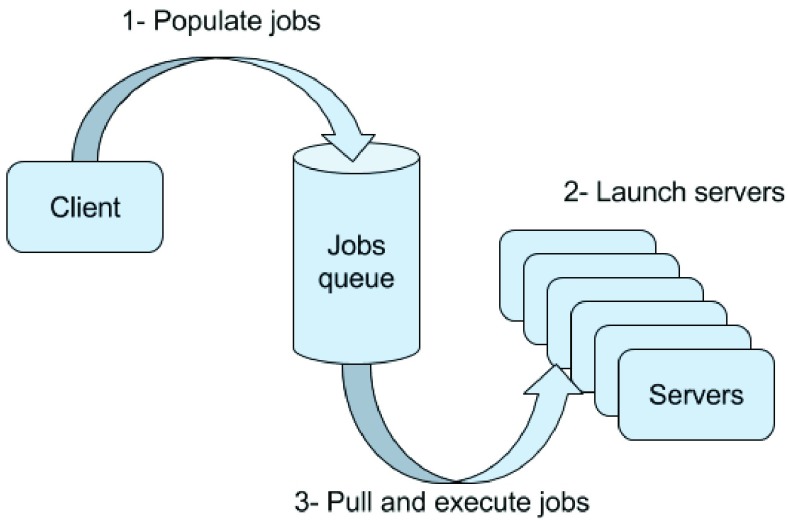
The cloud system setup and steps for acceleration of DIR.

The cloud system runs as follows. A master node (i.e., the client) populates the SQS queue with the jobs description and launches a number of EC2 nodes (i.e., the servers). After initialization, every EC2 executes a number of iterations where in every iteration, it pops a job message from the queue and executes it. This continues till there are no more jobs in the queue. These three steps are shown in Figure 3.

### E. Parameter Selection

As with any image registration algorithm, there are parameters in VS-GPU that must be set appropriately for a specific image registration task. A major advantage of executing the image registration and analysis on the cloud is having a relatively short turnaround time to get the results for a range of different parameter sets. This means that the parameters can be adjusted and fine-tuned in few hours at minimum cost. For our registration algorithm, we chose the parameters of interest as the ones with maximum expected influence on the performance of deformable registration of head and neck CT and CBCT images. The performance metrics for this parameter selection step were TRE. These parameters themselves were: (1) window/level [min, max] that is used to rescale intensities of the CBCT image into [0, 255] range, (2) minimum subvolume size at which to stop further subdivision, (3) degree of flexibility allowed for deformable registration, and (4) subdivision rate in the $z$ (superior-inferior) direction.

Window/level determines which subset of intensity range from the original image is rescaled and counted in the MI calculation. The MI calculation (or any such cost function calculation) is critical to the performance of an image registration algorithm so window/level is listed as one of the parameters to be optimized before applying the algorithm to CT-CBCT registration.

Subdivision in the algorithm stops when the size of subvolumes reaches a threshold minimum size. When the minimum subvolume size is set to be too large, subdivision stops too early that the registration may have not found the global optimal solution yet. When minimum subvolume size is too small, there may be a point of diminishing returns for the amount of computation relative to the deformation recovered.

Regarding the degree of flexibility parameter, the VS registration algorithm has a regularization term that restricts excessive movement of subvolumes. This parameter is a scalar, higher values of which represent more freedom for subvolumes to move and smaller values mean less allowable movement. Regardless of the setting of this parameter, image folding is never permitted in our implementation [20].

As for the subdivision rate in $z$, registration scenarios can include images with large and small axial coverages. In dealing with image data of our head and neck CT-CBCT application, the limited number of slices may make the dimensions of an individual subvolume significantly non-cubic, because the $z$ dimension is smaller than $x$ and $y$. Often this can lead to suboptimal registration results as subvolumes often explore their local solution space more effectively if they are cubic or near cubic. More cubic subvolumes can be constructed by not dividing along the z axis during the initial levels (i.e., dividing the initial volume into four subvolumes instead of eight). The subdivision rate specifies how many levels should be traversed before dividing in $z$.

Each parameter can be assigned a value from a set of predetermined values based on our own experience. Table 2 shows the parameter sets with predetermined values. Parameter set 1 consists of default values for all parameters and Parameter sets 2–12 were constructed by modifying the value of one parameter at a time.

**TABLE 2:** Parameter Sets Utilized to Optimize the CT-CBCT DIR

Parameter Set	Window3/Level	Minimum Subvolume Size (voxels)	Degree of Flexibility	Z Subdivision Parameter
1 (Default)	[−200, 900]	32	0	0
2	[−200, 900]	32	0	1
3	[−200, 900]	32	0	2
4	[−200, 900]	32	1	0
5	[−200, 900]	32	2	0
6	[−200, 900]	8	0	0
7	[−200, 900]	16	0	0
8	[−200, 800]	32	0	0
9	[−200, 800]	32	0	1
10	[0, 900]	32	0	0
11	[200, 900]	32	0	0
12	[200, 700]	32	0	0

**FIGURE 5.**
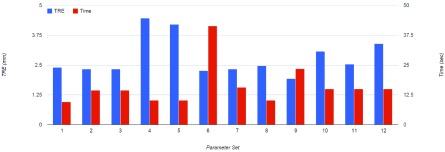
The plot of parameter optimization results from applying VS-GPU with each of 12 parameter sets to CT-CBCT registration in the training set for average TRE and the computation time.

The VS-GPU method implemented with each of the 12 parameter sets in Table 2 was applied to the training set (Cases 1–4 in Table 1). For each parameter set, there were 132 registration tasks, so $132 \times 12 =1$,584 registration tasks in total were performed for the 12 parameter sets. After registrations, TRE and computation time were used to identify the parameter set that led to the best performance of the VS-GPU as applied to head and neck CT-CBCT registration as shown in Figure 5. The DIR with the optimized parameter set was then applied to the remaining clinical cases for evaluation purposes.

### F. Comparison with Peer Registration Software

The VS-GPU method was compared with well- established image processing software packages, one available publicly (Elastix, version 4.6) and commercially (Velocity, version 2.8), based on the evaluation metrics described previously.

**FIGURE 4.**
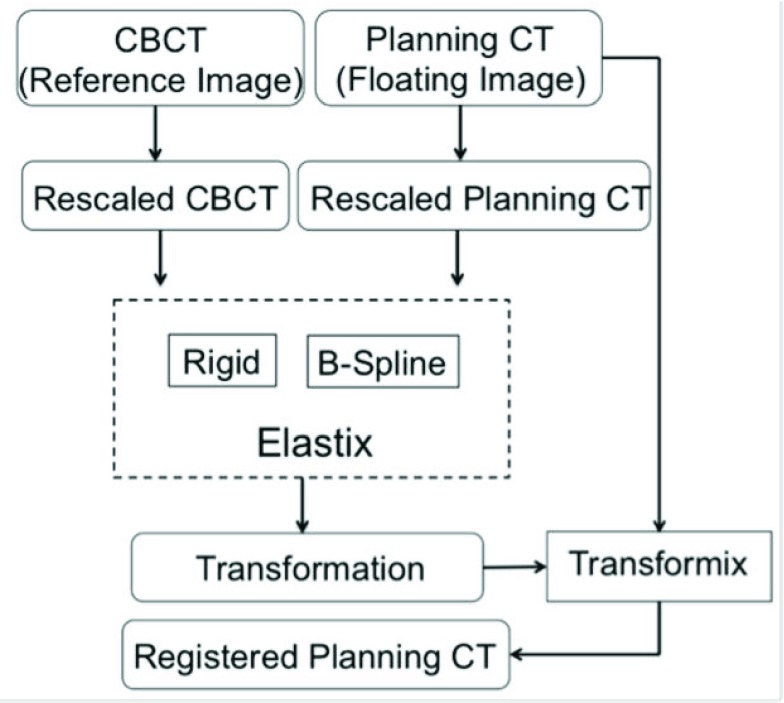
The flowchart showing application of Elastix to CT-CBCT DIR.

Elastix is an open-source software package developed using Insight Segmentation and Registration Toolkit (ITK). It is a comprehensive package with various options for registration methods and optimizations. As shown in Figure 4, we set Elastix to run rigid registration followed by a B-spline DIR with normalized MI [25] with regularization as the cost function. Regularization utilized the TransformRigidityPenalty to inhibit the transform search space. As with VS-GPU, we used the same parameter optimization framework to determine the weighting of this term relative to MI. Other parameters were set with recommended values as per the user manual. We noted that simply setting the regularization coefficient to a previously reported value, images were overwarped. The same intensity window/level as used for the VS-GPU method were used to preprocess planning CT and CBCT for the Elastix registration. Because we would like to compare speed of registration algorithms, the execution time for preprocessing and final warping (Transformix in Figure 4) was excluded from the reported computation time for Elastix.

Second peer software we selected was Velocity, which is commercially available and currently in clinical use in the Department of Radiation Oncology at the Johns Hopkins Hospital. To register the planning CT to daily CBCT, a volumetric region of interest was first chosen by the user. Rigid registration followed by multi-resolution B-spline DIR was performed. The outcome was the deformed planning CT that was registered to the daily CBCT along with a deformation vector field.

### G. Cloud-Based Quality Assessment

While the above validation metrics are appropriate for independent algorithm validation, many variables could affect registration quality for image pairs not included in the study. One of the benefits of relying on the cloud for computational resources is that we have the resources to add computationally intensive automatic quality assurance to every registration and still deliver these results at the time of care. To this end, we have added a consistency check of the registration results by performing a reverse registration. In other words, we verified that the CT to CBCT registration which we call the forward direction produces similar results to the CBCT to CT registration which we call the reverse direction. This verification is a necessary but not sufficient condition for the validity of the results and it can automatically detect and correct registration failures as follows. Consider a point $p$ in the physical space of the reference image (e.g., the CBCT fraction) to be transformed using the forward registration deformation field to obtain the transformed point $t$ in the CT physical space. Similarly $t$ is transformed using the reverse registration deformation field to produce $p'$ in the CBCT physical space. We call the physical distance between $p$ and $p'$ as the Selt TRE (STRE). Ideally if the registration is reversible the STRE should equal zero. Typical values for the STRE in case of correct registration result is less than a voxel size in mm. In our framework, we have calculate the STRE for approximately 1000 points inside the target region (e.g., around the isocenter or the calc point). If the average STRE value for these points is greater than the voxel size, registration is recalculated using different parameters.

## IV. Results

The results of parameter optimization with the training set are presented in Figure 5, which shows the average TRE and computation time from applying the VS-GPU method with each parameter set listed in Table 2 to the training set. Parameter set #9 was selected as the optimal set for continuing evaluation of VS-GPU on the test set (Cases 5–9).

**FIGURE 6.**
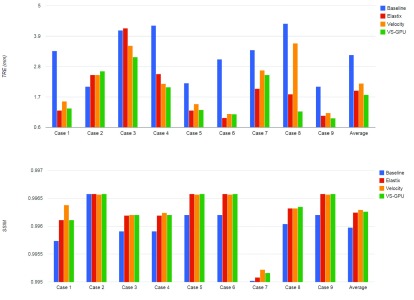
Average TRE valuation for methods VS-GPU, Elastix, Velocity, and before registration (Baseline).

**FIGURE 7.**
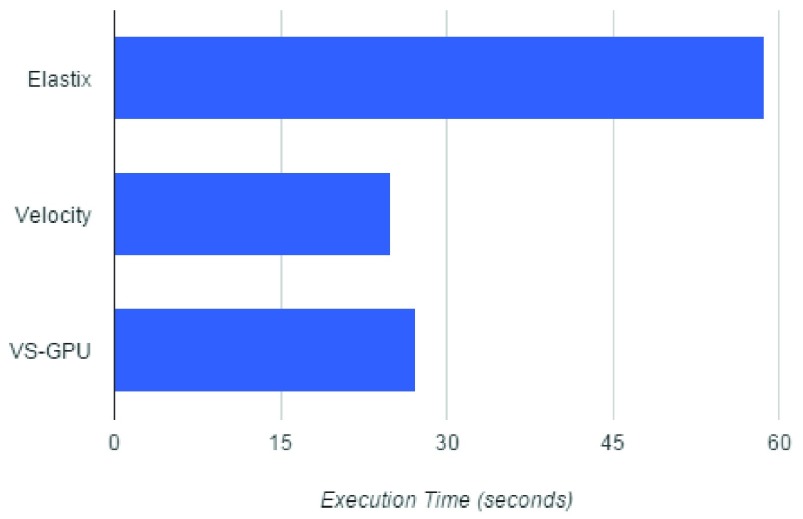
The average computation time for three different methods.

Results of applying the parameter optimized VS-GPU DIR method in comparison with Elastix and Velocity are shown in Figure 6. The figure shows SSIM index values for different methods. From the figure we observe that in all three methods led to improved similarity, with VS-GPU providing the best improvement, which indicates registered CTs in VS-GPU had the highest similarities with reference CBCTs measured by SSIM with relatively smallest standard deviations. Figure 7 shows the computation time. Velocity was estimated to finish a single registration in approximately 25s, whereas the time for VS-GPU and Elastix were recorded by CPU clock. Execution time of VS-GPU is nearly as fast as Velocity and almost twice as fast compared with Elastix.

The overall average TRE of VS-GPU across the entire test data set comprising 161 registrations was 1.8 ± 1.0 mm, whereas it was 1.9 ± 1.0 mm for Elastix, 2.2 ± 0.9 mm for Velocity.

**FIGURE 8.**
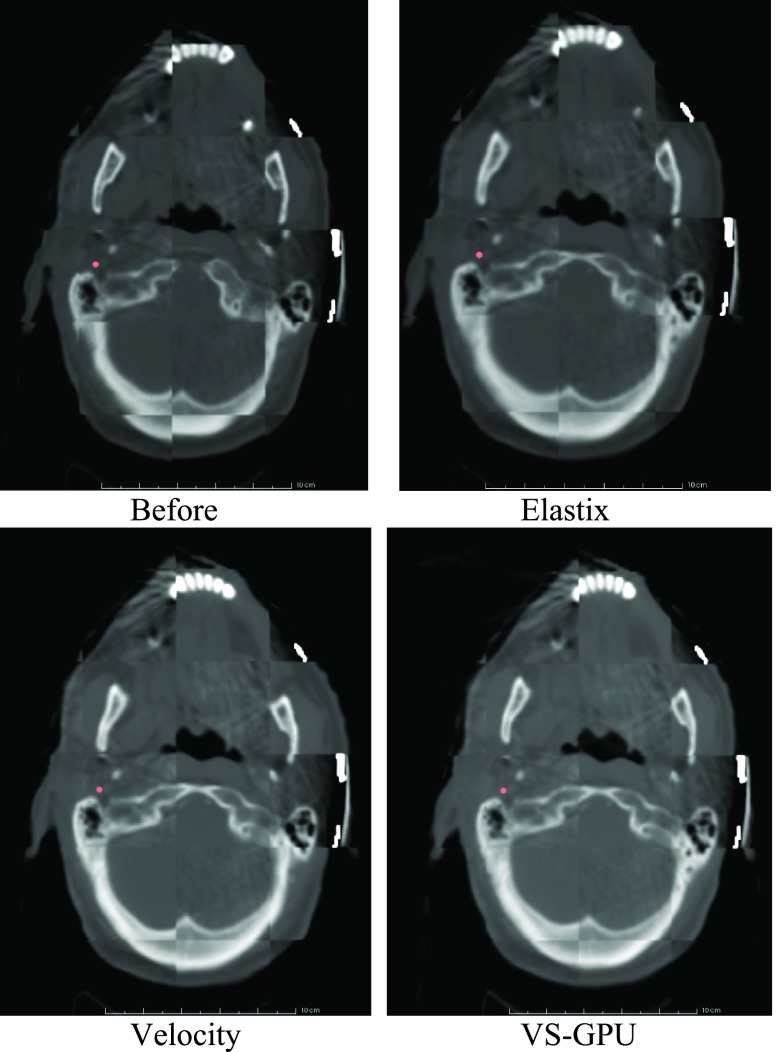
The visual comparison of VS-GPU, Elastix, and Velocity registration results with a checkerboard pattern. “Before” shows the reference and floating images with no registration. The red dot shows the landmark used for bed positioning.

The CT-CBCT registration for the last fraction of Case 8 was chosen to visually compare the three methods (VS-GPU, Velocity, Elastix), as shown in Figure 8. The reason for showing Case 8 was that it had the largest baseline TRE, which indicated that there existed a large starting misalignment between CBCT and planning CT. This was also the most challenging case among Cases 5–9 for image registration. A checkerboard display was generated with the reference image (CBCT) and the registered planning CT per each method. The exception was “Before,” which was generated from the reference CBCT and the original planning CT before any registration. All images in Figure 8 originated from the axial slice that contained CALC.

## V. Discussions

We have presented a GPU-accelerated DIR method: VS-GPU. The method is based on a previously reported volume subdivision-based registration algorithm that has been validated for various multimodality image registration applications [20]–[23]. The algorithm is locally rigid but globally deformable. Improved efficiency or accuracy compared with other DIR algorithms is an advantage of this algorithm. The algorithm is amenable to significant acceleration through hardware implementation. Since subvolumes of a given level may be registered independently, we are able to use coarse-grain parallelism in the algorithm to span multiple processing blocks. The MI calculation was accelerated using low-level data parallelism in the GPU, which enables the computation of MI to be orders of magnitude faster than a CPU only implementation.

Our VS-GPU is able to track deformable anatomic changes existing between per-fraction CBCTs and the planning CT and finish registration in less than a minute. This article focused on evaluating the accelerated implementation of the algorithm in the application of CT-CBCT DIR for radiation therapy of head and neck cancer patients. High-speed and accurate DIR could enable new applications, including margin optimization, which could have a profound impact on patient care. This study into the accuracy and speed of VS-GPU is a necessary first step for developing such applications.

Several approaches to speed up DIR have been reported [2]. One common approach is to perform registration in a multiresolution fashion where the solution of the coarser level is up-sampled and is used to initialize the next finer level. Another approach is to use hardware acceleration, mapping application parallelism to hardware concurrency. VS-GPU leverages both of these. The deformation field is calculated hierarchically in subdivision levels and, at each level, the transformation for each subvolume is calculated independently, which is convenient for introducing hardware acceleration.

With the increasing integration of high-performance graphic cards into affordable computers, GPU acceleration for DIR has been a topic of tremendous interested [26], [27]. In particular, Demons deformable image registration has been implemented with GPU acceleration by some groups [28], [29]. However, in published research on the effort of extending hardware acceleration, limited reporting can be found on multimodality DIR combined with MI as the similarity measure. Shams and Barnes proposed accelerated MI computation using GPU [30], and the team further implemented the GPU-accelerated MI computation into 3D medical image registration [31], but only with rigid registration.

We have presented the VS-GPU method, which has the implementation of the entire registration algorithm in graphics cards. It is a fast and fully automated multimodality DIR. Our experiments show that average execution time for registering two images with a volume size of $512 \times 512 \times 144$ voxels is less than 30 seconds. To demonstrate that the VS-GPU does not achieve such speeds at the expense of quality, we compared the registration accuracy with that of two peer methods using two different metrics. Results show that VS-GPU also demonstrates comparable accuracy.

TRE is a well-known metric to evaluate the accuracy of image registration. Normally a ground truth has to be established by marking corresponding points in both reference and floating images, which is usually performed by experts. We utilized the documented CALC to calculate TRE. In accordance with ICRU (International Commission of Radiation Units and Measurements) Report 50, reference point for reporting dose should be clinically relevant and placed inside the PTV. That reference point is CALC in our data sets.

Because our TRE evaluates one point by comparing couch shift with registration methods a second metric—SSIM index—was included to further evaluate the registration accuracy. The SSIM index measures the similarity of two images based on anatomical structures, instead of absolute intensity values, so it can be used for multimodality images such as CT and CBCT. TRE and SSIM index evaluate registration accuracy of VS-GPU from two different aspects. TRE focuses on point-based geometrical evaluation at CALC, which is clinically critical for dose calculation and monitoring, while SSIM measures the overall similarity between reference and registered images within the field of view of CBCT. The combination of TRE and SSIM thus provide a rigorous evaluation of the registration accuracy.

We compared our optimized algorithm with Elastix and Velocity. Velocity executes quickly and its results are clinically acceptable for existing applications of registration, but both Elastix and VS-GPU are able to outperform it in accuracy. Elastix results are nearly as accurate as VS-GPU, but requires twice as long to compute. VS-GPU therefore finds a niche in being fast and accurate, both critical traits for analyzing large datasets. Such large dataset analysis is the future direction of our work. With this foundation, the tumor and other critical structures motions can be monitored and statistically analyzed (for example, maximum and minimum movement along each axis) and this data can be incorporated into a future margin optimization algorithm.

## VI. Conclusion

We have presented VS-GPU, a high-speed and high-accuracy DIR method that can be applied to head and neck radiotherapy that can be readily accelerated with cloud computing and even assessed for quality on the cloud. We validated our method using clinical planning CT and CBCT data. We compared our registration method to Elastix, a widely used open-source method, and Velocity, a commercial registration method. Results showed that our method was twice as fast as Elastix with comparable accuracy. Results further showed that our method and the Velocity method offered comparable speed, but VS-GPU provided superior accuracy. Accuracy and speed are critical attributes for analyzing large datasets. As a future direction of our research, VS-GPU promises to serve as an enabling tool for a planned tumor margin optimization study based on large data analysis.
